# A case report: enhanced somatostatin receptor expression in metastatic pancreatic neuroendocrine tumor following everolimus therapy

**DOI:** 10.3389/fcell.2025.1658256

**Published:** 2025-10-24

**Authors:** Pei Zhang, Chenyan Zhang, Huanji Xu, Dan Cao

**Affiliations:** Division of Abdominal Tumor Multimodality Treatment, Cancer Center, West China Hospital, Sichuan University, Chengdu, Sichuan, China

**Keywords:** somatostatin receptor (SSTR), metastatic pancreatic neuroendocrine tumor, everolimus, SSA, PRRT

## Abstract

Pancreatic neuroendocrine tumors (pNETs) are rare and heterogeneous. Well-differentiated G1/G2 pNETs typically express somatostatin receptors (SSTRs), making them responsive to somatostatin analogue (SSA) therapy. However, therapeutic options become limited once SSTR expression decreases. This case report describes a 55-year-old man with grade 2 pNET who developed multiple liver metastases after undergoing pancreaticoduodenectomy in 2015. From August 2019 to October 2020, he received long-acting octreotide and transarterial chemoembolization (TACE), achieving stable disease. However, in August 2022, MRI scans indicated disease progression, leading to discontinuation of octreotide. In September 2022, oral surufatinib was initiated but paused in September 2023 due to adverse effects. In January 2024, everolimus therapy was started, resulting in a partial response by April 2024, with a significant reduction in liver metastases. Due to small intestinal ulcers, the dose of everolimus was reduced in August 2024. Follow-up scans showed stable disease through January 2025. In February 2025, [^68^Ga]Ga-DOTATATE PET/CT scans revealed significant re-expression of SSTR2 in liver lesions, likely induced by everolimus, allowing reinitiation of SSA therapy with increased octreotide dosage. This case demonstrates that everolimus can induce SSTR re-expression in advanced, SSTR-negative pNETs, offering new therapeutic possibilities. The “induction plus re-evaluation” approach could guide personalized treatment strategies in late-stage pNETs, although further studies are needed to validate this approach.

## 1 Background

Pancreatic neuroendocrine tumors (pNETs) are rare, and highly heterogeneous solid tumors that originate from the endocrine cells of the pancreas. They are characterized by the ability to secrete various hormones or neuropeptides, and account for approximately 1%–2% of all pancreatic neoplasms (Sonbol et al., 2022). With the ongoing advances in molecular imaging, laboratory diagnostics, and clinical awareness, the detection rates of pNETs have increased in recent years. However, their pathogenesis remains incompletely understood and involves a complex interplay of molecular events, including dysregulated gene expression, chromosomal deletions, and tumor suppressor gene methylation (Karimi et al., 2025).

Somatostatin receptors (SSTRs) are G-protein-coupled receptors that mediate the inhibitory effects of somatostatin on hormone secretion and cell proliferation. Among the five subtypes, SSTR2 is predominantly expressed in neuroendocrine tumors NETs and is crucial for targeted therapies (Pencharz et al., 2018). Somatostatin analogs (SSAs), such as octreotide and lanreotide, bind to SSTR2 to control hormone-related symptoms and inhibit tumor growth. Clinical studies, including the PROMID and CLARINET trials, have demonstrated that SSAs or SSAs combined with transarterial chemoembolization (TACE) can significantly prolong progression-free survival in patients with well-differentiated NETs.

In well-differentiated G1/G2 pancreatic pNETs, SSTRs, particularly SSTR2, are highly expressed, making them ideal therapeutic targets for SSAs like long-acting octreotide. These therapies not only offer symptomatic relief but also exert antiproliferative effects. The PROMID trial demonstrated that octreotide (30 mg monthly) significantly prolonged PFS in patients with metastatic midgut NETs, from 6 months (placebo) to 14.3 months (HR 0.34) (Rinke et al., 2009). Similarly, the CLARINET trial showed that lanreotide (120 mg every 4 weeks) improved PFS in patients with well-differentiated enteropancreatic NETs, the median PFS was not reached in the lanreotide group, compared to 18.0 months in the placebo group (HR 0.47) (Caplin et al., 2014). And SSAs are generally well-tolerated, with common side effects including gastrointestinal symptoms, hyperglycemia, and injection site reactions, serious adverse effects like cholelithiasis and bradycardia are rare.

For patients with liver metastases, the combination of SSA and transarterial chemoembolization (TACE) has been demonstrated to significantly prolong progression-free survival (PFS) and improve objective response rates, particularly in those with a high hepatic tumor burden (Megdanova-Chipeva et al., 2020).

Additionally, the anti-angiogenic inhibitor surufatinib or the mTOR inhibitor everolimus are firstly considered for advanced SSTR-negative pNETs and for SSTR-positive pNETs when disease progresses following SSA therapy. The SANET-p phase III trial demonstrated that surufatinib extended the median PFS from 3.7 to 10.9 months, while everolimus improved median PFS from 4.6 to 11.0 months in the RADIANT-3 study (Xu et al., 2020; Yao et al., 2016). Despite their efficacy, these targeted agents are frequently associated with adverse effects—including hypertension, proteinuria, oral ulcers, and bone marrow suppression—necessitating careful dose adjustment or treatment interruption based on individual tolerance (Herrera-Martínez et al., 2019).

Despite the progress made in treating NET with SSA, surufatinib, and everolimus, patients still face significant challenges after disease progression (Shi and Morse, 2022). There is a lack of standardized treatment options, and for those who progress after multiple lines of therapy, the prognosis is generally poor with limited survival (Mollazadegan et al., 2022).

Recent findings reveal that everolimus can enhance the expression of the SSTR2 gene and protein in NET cell lines *in vitro*, offering new therapeutic prospects for lesions initially lacking SSTR expression (von Hessert-Vaudoncourt et al., 2024). Building on this “induction and reassessment” strategy, advanced SSTR-negative G2 pNET patients may first receive everolimus to restore receptor expression, followed by [^68^Ga]Ga-DOTATATE imaging to determine renewed eligibility for peptide receptor radionuclide therapy (PRRT) or SSA treatment. This approach may expand therapeutic options and potentially improve survival outcomes.

This case report highlights the clinical significance of everolimus-induced SSTR re-expression, leading to the reintroduction of SSA. It underscores the value of multidisciplinary care and dynamic pathological monitoring in pNET management and offers a meaningful reference for personalized treatment strategies in advanced disease.

## 2 Case presentation

A 55-year-old man presented with scleral icterus and weight loss. In August 2015, contrast-enhanced abdominal CT revealed a 4.5 cm × 3.4 cm soft-tissue mass at the pancreaticoduodenal junction with marked enhancement. He underwent pancreaticoduodenectomy with cholecystectomy; histopathology confirmed a pNET G2, with immunohistochemistry showing PCK (+), CD56 (+), CgA (+), Syn (+), somatostatin (−) and a Ki-67 index of ∼5%.

At the follow-up on 15 July 2019, contrast CT of the chest and abdomen demonstrated multiple arterial phases enhancing nodules in the liver and enlarged lymph nodes along the superior mesenteric artery and mesentery, suggestive of metastases. Percutaneous liver biopsy confirmed metastatic neuroendocrine tumor, with tumor cells positive for PCK (+), CD56 (+), Syn (+), CgA (+), SSTR2 (+) and a Ki-67 index of ∼2%. Physical examination was negative for flushing, rash, edema, or acromegaly. Laboratory studies showed hemoglobin 68 g/L; liver and renal function, tumor markers (AFP, CEA, CA 19-9, CA 125, NSE), stool and urine tests were unremarkable. He had a 10-year history of hypertension, well controlled on medication. Multidisciplinary team (MDT) staging was pT3N1M0 (stage IIIB) pNET with multiple liver metastases (stage IV), suspected abdominal lymph-node involvement and, moderate anemia.

**FIGURE 1:**
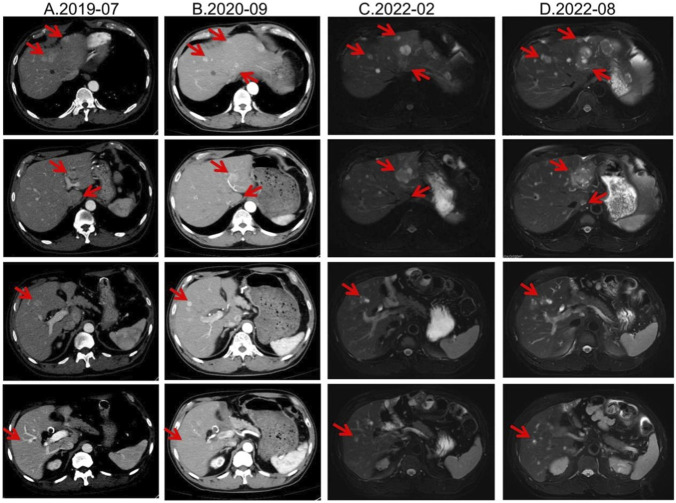
Lesion changes in patients treated with first-line SSA. **(A)** Multiple arterial-phase hyper-enhancing nodules are observed within the liver. The largest, located in the posterior-inferior segment of the right hepatic lobe, measures approximately 2.58 cm × 1.8 cm (2019-07). **(B)** After 15 cycles of treatment, multiple enhanced nodules were observed in the liver in the arterial phase, which were larger in the left lateral lobe of the liver, and the enhancement was reduced in the portal phase. Treatment response evaluation: SD (2020-09). **(C)** At the 33rd treatment cycle, the lesion in the liver showed marginal enhancement. The larger one was located in the left lateral lobe of the liver. Treatment response evaluation: SD (2022-02). **(D)** During the 40-cycle treatment, the lesions in the liver increased significantly compared with before. The larger ones were in the left lateral lobe of the liver, which was significantly larger than before. Treatment response evaluation: PD (2022-08).

From August 2019, he received long-acting octreotide 30 mg every 4 weeks as first-line systemic therapy, combined with five sessions of transarterial chemoembolization (TACE) between 20 August 2019 and 30 October 2020. From December 2019 to February 2022, all response evaluations indicated stable disease (SD). No significant adverse events were observed; anemia improved to ∼110 g/L with iron supplementation. The August 2022 MRI showed an increased size of hepatic lesions; treatment response was assessed as progressive disease (PD), and octreotide microspheres were discontinued (Figure 1).

**FIGURE 2:**
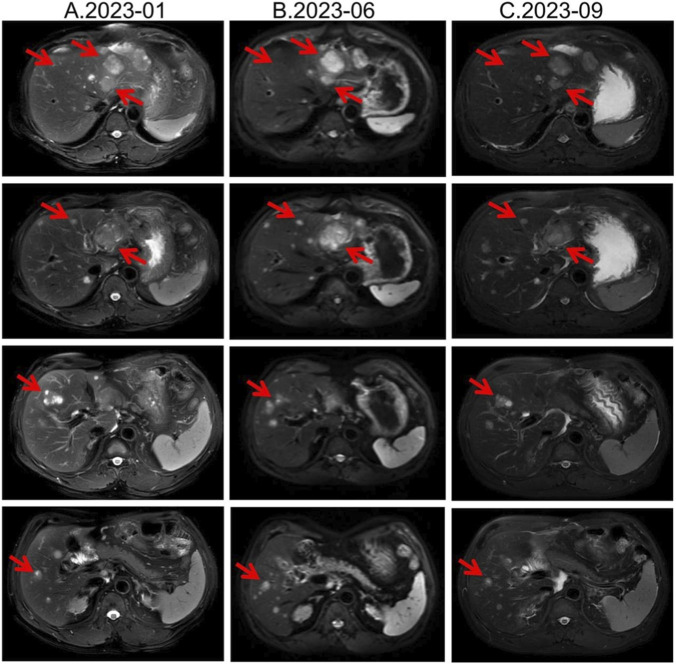
Lesion changes in patients treated with second-line surufatinib. **(A)** 2023-01 MRI: Multiple enhanced nodular shadows in the liver, with the larger ones located in the left lateral lobe of the liver, approximately 4.4 cm × 3.5 cm in size. Treatment response evaluation: SD. **(B)** 2023-06 MRI: Multiple enhanced nodules in the liver, with the larger ones located in the left lateral lobe of the liver. Treatment response evaluation: SD. **(C)** 2023-09 MRI: Multiple enhanced nodules in the liver, with the larger ones located in the left lateral lobe of the liver. Treatment response evaluation: SD.

**FIGURE 3:**
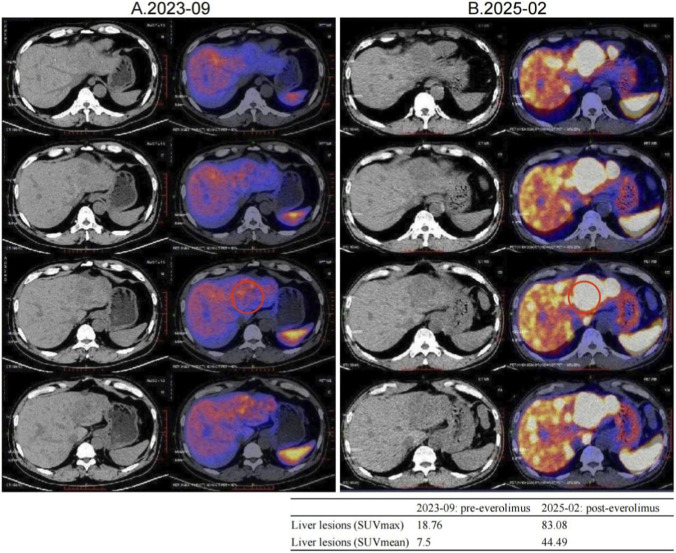
Changes in [^68^Ga]Ga-DOTATATE uptake were noted before and after everolimus, with a representative liver lesion highlighted by a red circle on both PET/CT panels. A small inset table summarizes its SUVmax and SUVmean values pre- and post-treatment. **(A)** 2023-09 [^68^Ga]Ga-DOTATATE PET/CT, Intravenous injection of [^68^Ga]Ga-DOTATATE (4.15 mCi), with whole-body imaging performed at approximately 60 min post-injection. Multiple slightly low-density shadows were seen in the liver parenchyma, and no Ga68 DOTATATE uptake was observed in some lesions within the liver. No increased uptake signals were observed in the abdominal and pelvic cavities. **(B)** 2025-02 [^68^Ga]Ga-DOTATATE PET/CT. Intravenous injection of [^68^Ga]Ga-DOTATATE, 3.13 mCi, followed by whole-body PET/CT imaging at approximately 60 min post-injection. Multiple liver metastases, with a significant increase in Ga68 DOTATATE uptake level compared to 2023-09 PET/CT (SUVmax:83.08). No increased uptake signals were observed in the abdominal and pelvic cavities.

Second-line therapy began in September 2022, with surufatinib 300 mg daily. In the SANET III phase III trial, surufatinib significantly prolonged PFS in pNETs (Xu et al., 2020). The MRI efficacy evaluations in January, June, and September 2023 all demonstrated SD (Figure 2). During treatment he developed mild-to-moderate proteinuria and anemia (hemoglobin 78 g/L). Considering the patient experienced intermittent melena in September 2023, after MDT discussion and temporary drug discontinuations he was screened for the “[^177^Lu]Lu-DOTATATE Injection versus Long-Acting Octreotide Study”. [^68^Ga]Ga-DOTATATE imaging demonstrated that some hepatic metastases were SSTR-negative, and he therefore failed screening (Figure 3). Surufatinib was resumed at a reduced dose of 200 mg daily. On 8 January 2024, the patient passed approximately 200 mL of dark red stool. A gastroscopy revealed multiple small-intestinal ulcers at the gastroenteric anastomosis site (A1 stage, Forrest III). After a comprehensive evaluation of efficacy and safety, surufatinib was discontinued.

**FIGURE 4:**
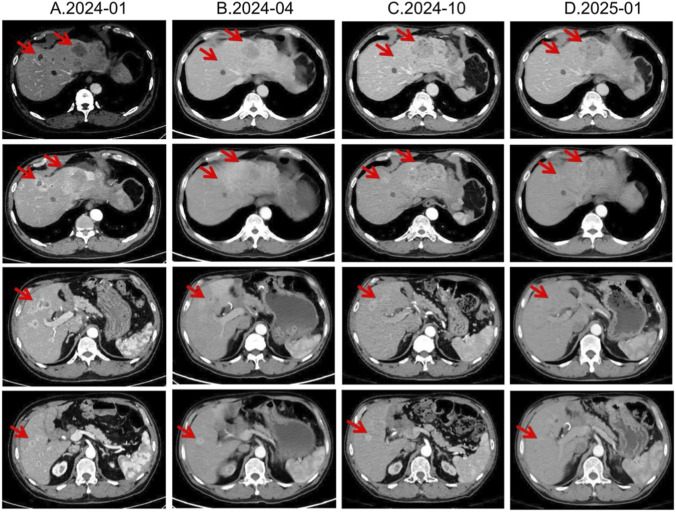
Lesion changes in patients during third-line everolimus treatment. **(A)** 2024-01 CT shows: Multiple weakly enhanced nodules in the liver, the largest of which is approximately 4.3 cm × 2.8 cm in size. **(B)** 2024-04 CT shows: The liver lesions have decreased and shrunk compared with most of the previous lesions. Treatment response evaluation: PR. **(C)** 2024-10 CT shows: Multiple slightly low-density nodular mass shadows in the liver, especially in the left outer lobe of the liver. Enhanced scans show uneven enhancement. Treatment response evaluation: SD. **(D)** 2025-01 CT shows: Multiple slightly low-density nodular mass shadows in the liver, especially in the left outer lobe, and enhanced scans show heterogeneous enhancement. Treatment response evaluation: SD.

In January 2024, third-line treatment with everolimus 10 mg daily was initiated. Everolimus, an oral mTOR inhibitor, has been shown in RADIANT-3 and RADIANT-4 to significantly delay progression of advanced pNETs (Yao et al., 2016). In April and July 2024, CT scans demonstrated shrinkage of most hepatic lesions, with the treatment response assessed as a partial response (PR). However, owing to multiple small-intestinal ulcers at that time, the everolimus dose was reduced to 5 mg once daily in August 2024. Subsequent CT re-evaluations in October 2024 and January 2025 both showed SD (Figure 4).

The MDT recommended repeating [^68^Ga]Ga-DOTATATE imaging to reassess tumor burden and SSTR2 expression. On 12 February 2025, [^68^Ga]Ga-DOTATATE uptake in multiple hepatic metastases was markedly increased, indicating SSTR2 upregulation following everolimus therapy (Figure 3; Supplementary Tables S1, S2). The patient was then re-screened for the “[^177^Lu]Lu-DOTATATE Injection versus Long-Acting Octreotide Study” and was randomized to the control arm, receiving 60 mg of long-acting octreotide every 4 weeks, and a CT performed in May 2025 showed SD on response assessment (Supplementary Figures S1, S2).

## 3 Discussion

First-line therapy for advanced pNETs typically includes SSAs to control tumor proliferation and symptoms, with optimal efficacy in SSTR-positive grade 1/2 NETs (Caplin et al., 2014; Del Rivero et al., 2023). Upon disease progression, the patient received surufatinib and everolimus. Surufatinib, a multikinase inhibitor targeting VEGFR and other receptors, suppresses angiogenesis and tumor growth (Huang et al., 2010). In the SANET-p phase III trial, surufatinib significantly improved PFS in pNETs (Xu et al., 2020). However, common adverse events—hypertension and proteinuria—require close monitoring; this patient’s surufatinib discontinuation underscores interpatient variability in tolerability. Following surufatinib cessation, everolimus was chosen as third-line therapy. In RADIANT-3, everolimus extended median PFS in advanced pNETs from 4.6 to 11.0 months (Yao et al., 2016). Our patient achieved a PR with everolimus, demonstrating both efficacy and tolerability.

Notably, this patient exhibited dynamic changes in SSTR expression during everolimus treatment. Pre-treatment [^68^Ga]Ga-DOTATATE imaging showed negligible uptake, indicating low SSTR expression often seen in poorly differentiated or highly proliferative NETs (Kim et al., 2024). After several months of everolimus, hepatic lesions regained high SSTR expression and “lit up” on imaging—a phenomenon of SSA receptor re-expression that has gained attention (Mileva et al., 2021).

Quantitatively, although SUV_max values of 10–40 are common in well-differentiated NETs, outliers with very high uptake have been reported on [^68Ga]Ga-DOTATATE PET/CT (tumor SUV_max up to 118), so the value of 83.08 observed here falls within published extremes (Kayani et al., 2009). Our two studies were acquired on the same system with ∼60-min uptake times and similar activities, consistent with current SNMMI/EANM procedure standards; thus, protocol differences are unlikely to explain the increase (Hope et al., 2023). The marked rise is more plausibly attributed to high post-treatment SSTR2 availability and favorable background activity (with normal spleen and liver SUVs in the expected ranges), as reflected by elevated tumor-to-liver and tumor-to-spleen ratios now detailed in Supplementary Tables S1, S2.

Beyond mTOR inhibitors, case reports suggest that CAPTEM (capecitabine + temozolomide) chemotherapy may similarly induce SSTR re-expression. One study described increased SSTR expression post-CAPTEM and explored mechanisms involving cell‐cycle alterations and DNA damage responses (Sharma and Basu, 2020). Collectively, these findings point to a “receptor restoration” phenomenon: systemic therapies can render initially SSTR-negative tumors receptor-positive, offering new therapeutic opportunities for refractory NET patients.

Although we could not obtain post-everolimus tumor tissue to confirm SSTR2 re-expression directly, several compelling lines of evidence support its plausibility. In bronchopulmonary NET lines (H720/H727), combined PI3K/mTOR inhibition with low-dose everolimus increased SSTR2 mRNA and immunoreactive score by > 2-fold, paralleled by heightened lanreotide sensitivity (von Hessert-Vaudoncourt et al., 2024). Earlier pancreatic-NET work showed that adding nanomolar everolimus to BYL-719 boosted SSTR2 transcription 12-fold in BON-1 and 1.5-fold in QGP-1 cells, quantified by RT-qPCR (Nölting et al., 2017). Murine xenograft models likewise displayed higher tumor-to-background ratios on [^68^Ga]Ga-DOTATATE PET after everolimus, consistent with drug-induced SSTR2 gene upregulation (Zellmer et al., 2022). A multi-tumor xenograft model showed that everolimus pretreatment markedly increased cell-surface GPCR density, boosting radiolabeled minigastrin uptake and prolonging survival after PRRT (Grzmil et al., 2022). Collectively, these studies justify prospective evaluation of an “everolimus-induction →SSA/PRRT” sequence and frame our single-patient observation within an emerging body of molecular evidence.

Current guidance agrees that PRRT with [^177Lu]Lu-DOTATATE is appropriate for metastatic or unresectable NETs provided the tumor is SSTR-positive on imaging (National Comprehensive Cancer Network, 2025; Pavel et al., 2020). Importantly, none of the major guidelines or labels list “re-expressed” SSTR as a contraindication. Absolute contraindications include pregnancy or acute unstable illness, whereas relative contraindications primarily involve severe renal or bone marrow involvement. In our patient, renewed SSTR2 positivity met the imaging criterion, and there were no biological or safety-based barriers to PRRT—the choice of SSA instead reflected adherence to the randomized trial protocol. The phase III COMPETE trial directly compared [^177Lu]Lu-edotreotide versus everolimus in SSTR-positive G1/2 GEP-NETs, demonstrating superior efficacy and safety for PRRT (median PFS 23.9 vs. 14.1 months) (Author Anonymous, 2025). The present case suggests that an “induction + re-evaluation” approach—using everolimus to upregulate SSTR followed by repeat imaging—could allow PRRT or SSA rechallenge in patients originally deemed ineligible, thereby extending treatment options.

However, everolimus-induced re-expression of SSTR2 is supported only by preclinical work and a handful of small case series, including the present report. As a single-patient observation, our data are subject to selection bias and cannot reflect the biological and clinical heterogeneity of pancreatic neuroendocrine tumors. Variables such as tumor grade, molecular profile, prior treatments, and comorbidities may all modulate SSTR dynamics and treatment response. Rigorous validation will require prospective studies that serially measure SSTR2 gene and protein levels before and after everolimus and that test a sequenced “everolimus induction → PRRT/SSA” approach on survival outcomes in larger, well-characterised cohorts. If confirmed, such a strategy could shift NET management from passive tumor control toward deliberately priming tumors for more effective subsequent therapies.

## 4 Conclusion

In summary, this case underscores the importance of individualized, multidisciplinary management for pNETs and highlights the plasticity of tumor biology. Everolimus may upregulate SSTR expression, thereby creating a therapeutic window for receptor-targeted therapies in patients who were initially ineligible. Clinicians should therefore collaborate across specialties to develop personalized treatment plans and, when disease progresses, reconsider the tumor’s molecular imaging and biological profile. As the mechanisms underlying this “receptor re-expression” phenomenon are elucidated, we may be able to integrate it routinely into clinical practice and further improve outcomes for patients with neuroendocrine tumors.

## Data Availability

The original contributions presented in the study are included in the article/Supplementary Material, further inquiries can be directed to the corresponding authors.

## Abbreviations

[^68^Ga]Ga-DOTATATE, Gallium-68-labeled DOTA-D-Phe1-Tyr3-octreotate; [^177^Lu]Lu-DOTATATE, Lutetium-177-labeled DOTA-D-Phe1-Tyr3-octreotate; CAPTEM, Capecitabine + Temozolomide; CD56, Cluster of Differentiation 56; CgA, Chromogranin A; GEP-NETs, Gastroenteropancreatic Neuroendocrine Tumors; Ki-67, A protein associated with cell proliferation; mTOR, Mammalian Target of Rapamycin; MRI, Magnetic Resonance Imaging; PD, Progressive Disease; PFS, Progression-Free Survival; PCK, Pancreatic Cytokeratin; PR, Partial Response; PRRT, Peptide Receptor Radionuclide Therapy; SD, Stable Disease; SSTR2, Somatostatin Receptor Subtype 2; SSTRs, Somatostatin Receptors; SSAs, Somatostatin Analogs; TACE, Transarterial Chemoembolization; VEGFR, Vascular Endothelial Growth Factor Receptor.
