# Supplementary material for: A case report: enhanced somatostatin receptor expression in metastatic pancreatic neuroendocrine tumor following everolimus therapy
Source: Front Cell Dev Biol. 2025 Oct 24;13:1658256. doi: 10.3389/fcell.2025.1658256 (PMC12592188; doi:10.3389/fcell.2025.1658256)
Supplement: Supplementary file 1 [file Table1.pdf]

Supplementary Tables

SUVmax	pre-everolimus	post-everolimus
Spleen	28.25	20.08
Normal liver parenchyma	12.89	10.42
Liver lesions	18.76	83.08

Supplementary Table 1: Maximum standardized uptake values (SUVmax) of spleen, normal liver parenchyma, and liver lesions measured on [^{68}Ga]Ga-DOTATATE PET/CT before and after everolimus treatment. Values are presented as absolute SUVmax at each time point.
