# Supplementary material for: A case report: enhanced somatostatin receptor expression in metastatic pancreatic neuroendocrine tumor following everolimus therapy
Source: Front Cell Dev Biol. 2025 Oct 24;13:1658256. doi: 10.3389/fcell.2025.1658256 (PMC12592188; doi:10.3389/fcell.2025.1658256)
Supplement: Supplementary file 2 [file Table2.pdf]

SUVmean	pre-everolimus	post-everolimus
Spleen	20.3	17.96
Normal liver parenchyma	6.39	8.28
Liver lesions	7.5	44.49

Supplementary Table 2: Mean standardized uptake values (SUVmean) of spleen, normal liver parenchyma, and liver lesions measured on [^{68}Ga]Ga-DOTATATE PET/CT before and after everolimus treatment. Values are presented as absolute SUVmean at each time point.
