# Supplementary material for: A case report: enhanced somatostatin receptor expression in metastatic pancreatic neuroendocrine tumor following everolimus therapy
Source: Front Cell Dev Biol. 2025 Oct 24;13:1658256. doi: 10.3389/fcell.2025.1658256 (PMC12592188; doi:10.3389/fcell.2025.1658256)
Supplement: Supplementary file 3 [file Image2.pdf]

Supplementary Material

Supplementary Figures

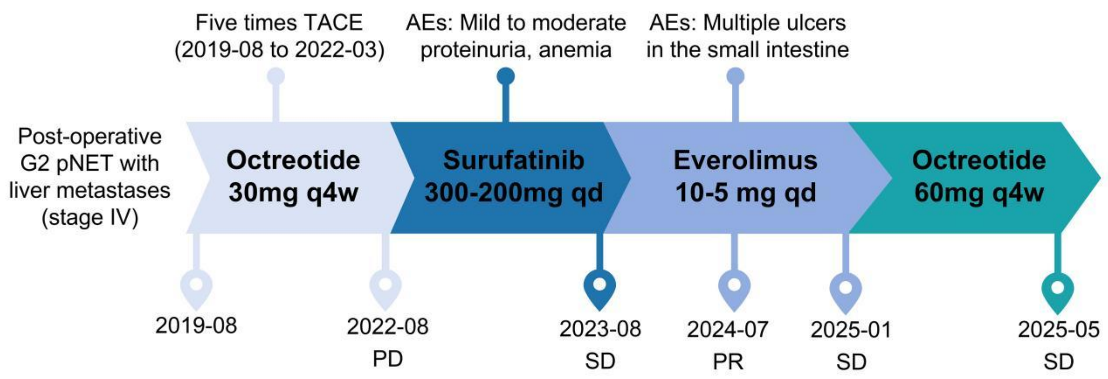

Supplementary Figure 2: Treatment flow chart of patients from August 2019 to May 2025. Since August 2019, this postoperative grade 2 pNET patient with stage IV liver metastases has received four consecutive lines of systemic therapy. First-line: Long-acting octreotide 30 mg every four weeks combined with hepatic-artery TACE from 2019-08 to 2022-08. Second-line: Surufatinib 300 mg once daily, later reduced to 200 mg once daily, from 2022-09 to 2023-09. Third-line: Everolimus 10 mg once daily, subsequently tapered to 5 mg once daily, from 2024-01 to 2025-01. Fourth-line: High-dose octreotide 60 mg every four weeks, initiated in February 2025; three cycles have been completed to date.
