# Supplementary material for: A case report: enhanced somatostatin receptor expression in metastatic pancreatic neuroendocrine tumor following everolimus therapy
Source: Front Cell Dev Biol. 2025 Oct 24;13:1658256. doi: 10.3389/fcell.2025.1658256 (PMC12592188; doi:10.3389/fcell.2025.1658256)
Supplement: Supplementary file 4 [file Image1.pdf]

Supplementary Material

Supplementary Figures

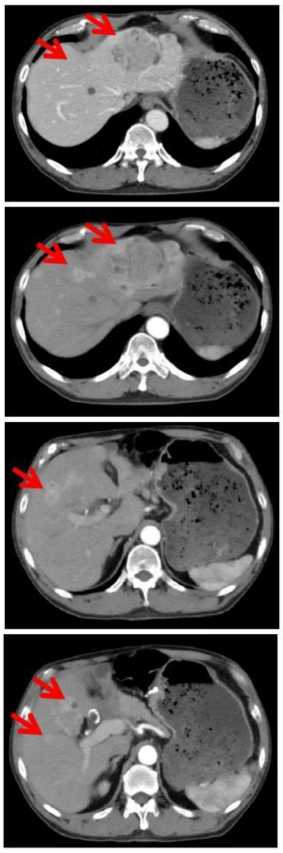

Supplementary Figure 1: Lesion changes in patients treated with three cycles of octreotide. Multiple enhanced nodular shadows in the arterial phase were observed in the liver, with the larger ones located in the left lateral lobe of the liver. Compared with the old films from 2025-01, the changes were not obvious. Treatment response evaluation: SD.
